# Technical Perspectives on Applications of Biologically Coupled Gate Field-Effect Transistors

**DOI:** 10.3390/s22134991

**Published:** 2022-07-01

**Authors:** Toshiya Sakata

**Affiliations:** Department of Materials Engineering, School of Engineering, The University of Tokyo, 7-3-1 Hongo, Bunkyo-ku, Tokyo 113-8656, Japan; sakata@biofet.t.u-tokyo.ac.jp; Tel.: +81-3-5841-1842

**Keywords:** biosensing, potentiometric biosensor, biologically coupled gate field-effect transistor (Bio-FET), ionic and biomolecular charge, Debye length, measurement solution, pH response, subthreshold slope, semiconductive material, integrated device

## Abstract

Biosensing technologies are required for point-of-care testing (POCT). We determine some physical parameters such as molecular charge and mass, redox potential, and reflective index for measuring biological phenomena. Among such technologies, biologically coupled gate field-effect transistor (Bio-FET) sensors are a promising candidate as a type of potentiometric biosensor for the POCT because they enable the direct detection of ionic and biomolecular charges in a miniaturized device. However, we need to reconsider some technical issues of Bio-FET sensors to expand their possible use for biosensing in the future. In this perspective, the technical issues of Bio-FET sensors are pointed out, focusing on the shielding effect, pH signals, and unique parameters of FETs for biosensing. Moreover, other attractive features of Bio-FET sensors are described in this perspective, such as the integration and the semiconductive materials used for the Bio-FET sensors.

## 1. Introduction

Ionic or biomolecular charges induce a change in potential at the electrolyte solution/electrode interface. As a type of potentiometric biosensor, biologically coupled gate field-effect transistors (Bio-FETs), which are originally based on solution-gated FETs, are attracting attention worldwide [1,2,3,4,5,6]. This is probably because various types of biomolecules with charges can be directly detected as electrical signals with the Bio-FETs in a label-free and real-time manner, and various semiconductive materials can also be applied to biosensing [7,8,9,10,11,12,13,14]. Furthermore, the integrated Bio-FET chip based on a complementary metal oxide semiconductor (CMOS) technology enables the simultaneous detection of multiple samples [15].

However, some critical issues constrain such advantages of the Bio-FETs, such as the shielding effect due to counter ions (Debye length limit) and the fabrication process. The Debye length limit is controlled by changing the ionic strength in a measurement solution, that is, diluted measurement solutions are useful for improving the detection sensitivity of the Bio-FETs to charged biomolecules because of the reduction in the shielding effect by counter ions [16,17,18,19,20,21,22,23,24,25,26,27,28,29,30]. Although the dilution of measurement solutions contributes to the improvement, it is not useful for real samples with high ionic strengths such as blood in a real-time measurement [30], depending on the application. On the other hand, solution-gated FETs are promising for the detection of changes in pH owing to the equilibrium reaction between hydrogen ions with the smallest size and hydroxy groups at an oxide gate insulator, in accordance with the Nernstian response. That is, the detection of changes in pH induced by biological phenomena may be straightforward and effective for biosensing with solution-gated FETs [31,32,33,34,35,36,37,38,39,40,41], although various receptor molecules should be modified on the gate electrode to specifically and selectively detect target biomolecules and to broaden the applications of Bio-FETs as a platform technology for biosensing, considering the Debye length limit.

Moreover, the Bio-FETs are not simple potentiometric biosensors. In other words, their features can be effectively utilized for biosensing. For instance, the subthreshold slope (SS) near the thermal limit contributes to a large shift in drain current (*I*_D_) at a constant gate voltage (*V*_G_) in the SS region, indicating a high sensitivity with a low limit of detection (LOD) [14,42,43]. Alternatively, the capacitive components of functional polymer membranes on the gate electrode are electrically changed by the interaction with noncharged biomolecules [41,44,45].

Considering the above, the technical issues of the Bio-FETs are pointed out in this perspective, focusing on the shielding effect, pH signals, and the unique parameters of FETs for biosensing.

## 2. How Is the Measurement Solution Used?

**Figure 1:**
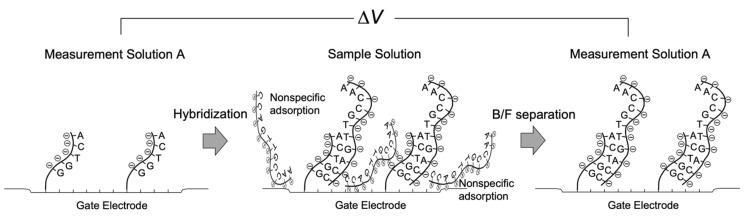
Schematic illustration of measurement process with Bio-FET.

Around two decades ago, a nonoptical and label-free DNA analytical method was proposed on the basis of Bio-FET technology [17,18,19,20,21,22,23,24,25,26,27,28]. Not only were DNA molecules an easy target for Bio-FETs owing to their molecular charges based on phosphate groups, but the development of label-free DNA chips was also actively pursued as one of the post-genome technologies. Single-stranded DNA probes were chemically tethered on the gate electrode, and then the complementary DNA targets were hybridized with the probes, the immobilization density of which was at least on the order of, ca., 10^11^/cm^2^ [26], inducing the change in the density of negative charges on the gate electrode (Figure 1). Moreover, extension reactions were performed for nonhybridized sequences of target DNA partly complementary to the probe on the gate electrode, resulting in the increase in the density of negative charges. Indeed, these reactions were successfully detected for DNA molecules with a few tens of bases on the basis of the principle of Bio-FETs, whereas longer DNA sequences could not be electrically detected [27]. However, relatively long DNA molecules of approximately 5–10 nm in length could be detected with the Bio-FETs as expected. This expectation was based on the detection of DNA molecular recognition events in a measurement buffer solution with a relatively low ionic strength (i.e., relatively large Debye length) after the bound/free (B/F) molecule separation for each reaction. That is, targeted molecules are specifically bound to substrates, whereas molecules nonspecifically and unexpectedly adsorbed there are washed out. Note that the same buffer solution should be used for each measurement after the B/F molecule separation because the effect of buffer concentration on signal drifts could be neglected. This means that the DNA chip for applications such as single-nucleotide polymorphism (SNP) genotyping, which is based on the hybridization or extension reaction, is tolerant to the B/F separation in every measurement. Thus, the diluted measurement solution can be used for reducing the shielding effect by counter ions. In addition, the B/F separation may be needed to wash out the gate electrode and reduce the nonspecific adsorptions of interfering species with charges. Then, the same measurement solution should be used before and after the reactions to maintain the Debye length. Their applications do not necessarily require the in situ measurement of real samples containing more counter ions. Similarly, the above consideration is also applicable to antigen–antibody reactions and so forth [29].

## 3. Straightforward Mechanism in Bio-FETs

**Figure 2:**
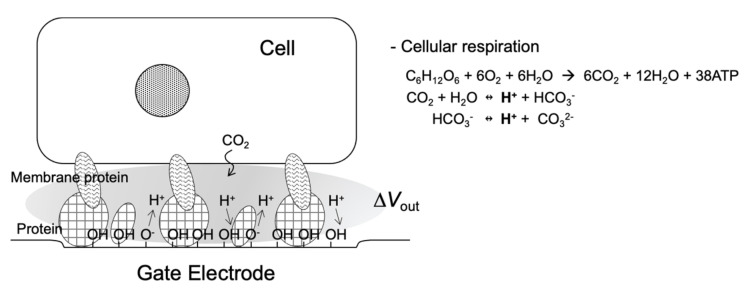
Schematic illustration of nanogap interface between cell and Bio-FET.

A general cell culture medium includes various ions and chemicals such as serum and glucose. As described in Section 2, in such a medium, the shielding effect caused by counter ions is a problem because Bio-FETs are very insensitive to the changes in the density of molecular charges based on biomolecular recognition events on the gate electrode in the cell culture medium. In other words, nonspecific electrical signals can be prevented from interfering with species in the cell culture medium because some proteins contained in it have been nonspecifically adsorbed on the gate electrode during preculture. Then, what specific targets are detected by the Bio-FETs under this condition? Hydrogen ions, in particular, which have the smallest size, induce changes in pH. Actually, cellular respiration activities can be easily and continuously monitored for any living cells using Bio-FETs with an oxide gate electrode in the cell culture medium [32,34,35,36,37,38,39,40]. Some proteins in the cell culture medium are adsorbed at the oxide gate surface during preculture, resulting in the adhesion of cells at the substrate. These macromolecules prevent targeted ionic charges from coming into contact with the gate, but hydrogen ions can easily attach to the oxide gate surface, where the equilibrium reaction between hydroxyl groups and hydrogen ions contributes to the change in the charge density at the oxide gate electrode (Figure 2). Moreover, hydrogen ions are concentrated in the closed nanogap space between the cell membrane and the oxide gate electrode [36,38]. This detection mechanism is very simple, that is, living cells are simply cultured on the oxide gate electrode of the original solution-gated FET (i.e., pH-responsive ion-sensitive FET (ISFET)) for monitoring cellular respiration, although there is a report that the action potential of nerve cells can be monitored in less than one second on the basis of the capacitive coupling model of the cell membrane and the oxide gate electrode [46]. In addition, the cell culture medium with high ionic strength contributes to the reduction in the effect of other ionic and biomolecular charges on the output signal by minimizing the Debye length. This is a straightforward mechanism in the pH-responsive ISFET. As a similar case, we had a breakthrough in label-free DNA sequencing with arrayed ISFET devices based on the CMOS process, which resulted in massively parallel DNA sequencing followed by a cost-effective and high-speed gene analysis [15]. This method was based on the detection of ionic charges, that is, not negative charges of extended base pairs mentioned in Section 2 but positive charges of hydrogen ions generated by enzymatic reactions as byproducts [31]. This means that the pH-responsive ISFET was principally utilized for label-free DNA sequencing, which makes the Debye length limit almost negligible. Thus, it is also important to reconsider the intrinsic features of Bio-FETs, which allow the stable monitoring without additional modifications of the gate electrode.

## 4. Features of Transistor for Biosensing

**Figure 3:**
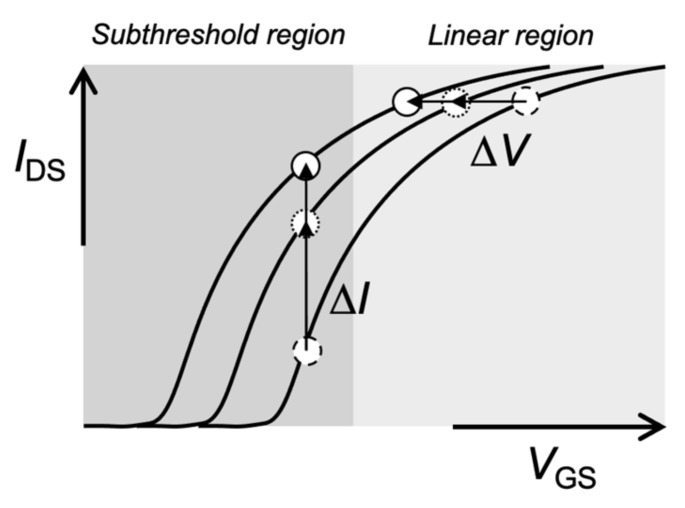
Schematic illustration of *V*_GS_–*I*_DS_ transfer curve of Bio-FET.

In general, biomolecular recognition events can be analyzed from transistor characteristics such as a *V*_G_–*I*_D_ transfer characteristic (e.g., Δ*V*_G_ at a constant *I*_D_ regarded as a threshold voltage shift (Δ*V*_T_)) (Figure 3). Mostly, Δ*V*_G_ at a constant *I*_D_ before and after various biomolecular recognition events (e.g., DNA hybridization) is estimated in the linear region of Bio-FETs. This evaluation method is appropriate for potentiometric biosensors. Indeed, pH-responsive ISFETs ideally show the Nernstian response (59.2 mV/pH at 25 °C) on the basis of Δ*V*_G_ at a constant *I*_D_ (Δ*V*_T_). On the other hand, ultrasensitive recognition of biomolecules is expected in the subthreshold regime of Bio-FETs (Figure 3). For instance, the solution-gated FET with a 20 nm thick indium tin oxide (ITO) channel exhibited a markedly steep SS, which was very close to the thermal limit (60 mV/dec at 300 K) and may result in a steep SS of less than 60 mV/dec in two-dimensional (2D)-FETs [14]. As a result, the electrical signals measured in the subthreshold regime were about 10 times larger than those measured in the linear regime, which could contribute to the ultrasensitive detection of biomolecules. Moreover, the sensitivity of one-dimensional (1D) nanowire-FET sensors was exponentially enhanced in the subthreshold regime [43]. Thus, the intrinsic features of Bio-FETs should be further improved for biosensing. Note that the Bio-FETs with steeper SS should also be developed not only as simple potentiometric biosensors, although their electrical stabilities have to be improved for the measurements in electrolyte solutions. With these features, 1D and 2D semiconductive materials (1D, e.g., silicon nanowire and carbon nanotube; 2D, e.g., graphene and molybdenum disulfide (MoS_2_)) are attractive for the development of novel Bio-FETs owing to their high responsiveness [7,8,9,10,11,14,43].

Moreover, Δ*V*_T_ in the solution-gated FETs is based on the change in the density of ionic and molecular charges at the gate electrode. As mentioned in Section 1, the equilibrium reaction between hydrogen ions and hydroxyl groups at the oxide gate electrode contributes to the change in the charge density at the gate electrode surface, which depends on pH. pH-responsive ISFETs with the oxide gate electrode (e.g., Ta_2_O_5_) ideally follow the Nernstian response because the site density of hydroxy groups at the Ta_2_O_5_ surface is expected to be about 10^15^/cm^2^ [47], which is sufficiently high. That is, regardless of the area of the oxide gate electrode, which comes in direct contact with electrolyte solutions, such pH-responsive ISFETs must show the Nernstian response with the change in pH if the change in the charge density is identical. In accordance with this concept, the smaller the area of the gate electrode, the fewer the number of biomolecules reacting at the gate electrode surface. This indicates that a single-biomolecule measurement may be realized using Bio-FETs with a smaller area of the gate electrode on a molecular scale. Actually, the nanowire-based Bio-FETs appear to show an ultrasensitive biomolecular recognition [7]. In addition, the pH responsivity may be increased beyond the Nernst limit using dual-gate FETs with nanowires on the basis of the capacitive coupling effect between the liquid and bottom gates [48,49,50]. Note that the amplification of electrical signals based on the detection principle may include that of background noise derived from interfering species, leakage, photoinduced fluctuations, and the temperature effect, as well as that of specific signals expected from targeted biomolecules. That is, some treatments such as surface modifications of functional membranes at the active gate electrode are required for increasing the signal-to-noise ratio (S/N).

## 5. Conclusions

In this perspective, the significant features of Bio-FETs and the important points for measuring using the Bio-FETs were indicated, focusing on the measurement solution, their basic and reliable pH dependence, and the transistor parameters. In addition, the arrayed-gate Bio-FETs should be necessarily applied for multibiosensing, as mentioned in Section 1. This may be actually the most unique feature of FETs because other biosensors (e.g., surface plasmon resonance (SPR) sensors and quartz crystal microbalance (QCM) sensors) hardly enable the integration of electrodes as in CMOS sensors. Moreover, FETs are commonly used in various electric devices such as smartphones and body thermometers because FETs in themselves are miniaturized and included in such devices. Moreover, new semiconductive materials, the functionalities of which are controlled on the nanometer order, must expand the possible applications of Bio-FETs in the future. Note that functional membranes at the electrolyte solution/gate electrode interface should be continuously developed for detecting selectively specific target biomarkers [51,52,53,54,55,56], considering the prevention/filtering of nonspecific signals based on interfering species [57,58]. Moreover, such functional membranes (e.g., lipid membrane) may extend the Debye length to improve the detection limit for biosensing [59].

## Data Availability

Not applicable.
